# Supplementary figures and images for: Freeze-drying can replace cold-chains for transport and storage of fecal microbiome samples
Source: PeerJ. 2022 Mar 15;10:e13095. doi: 10.7717/peerj.13095 (PMC8932309; doi:10.7717/peerj.13095)

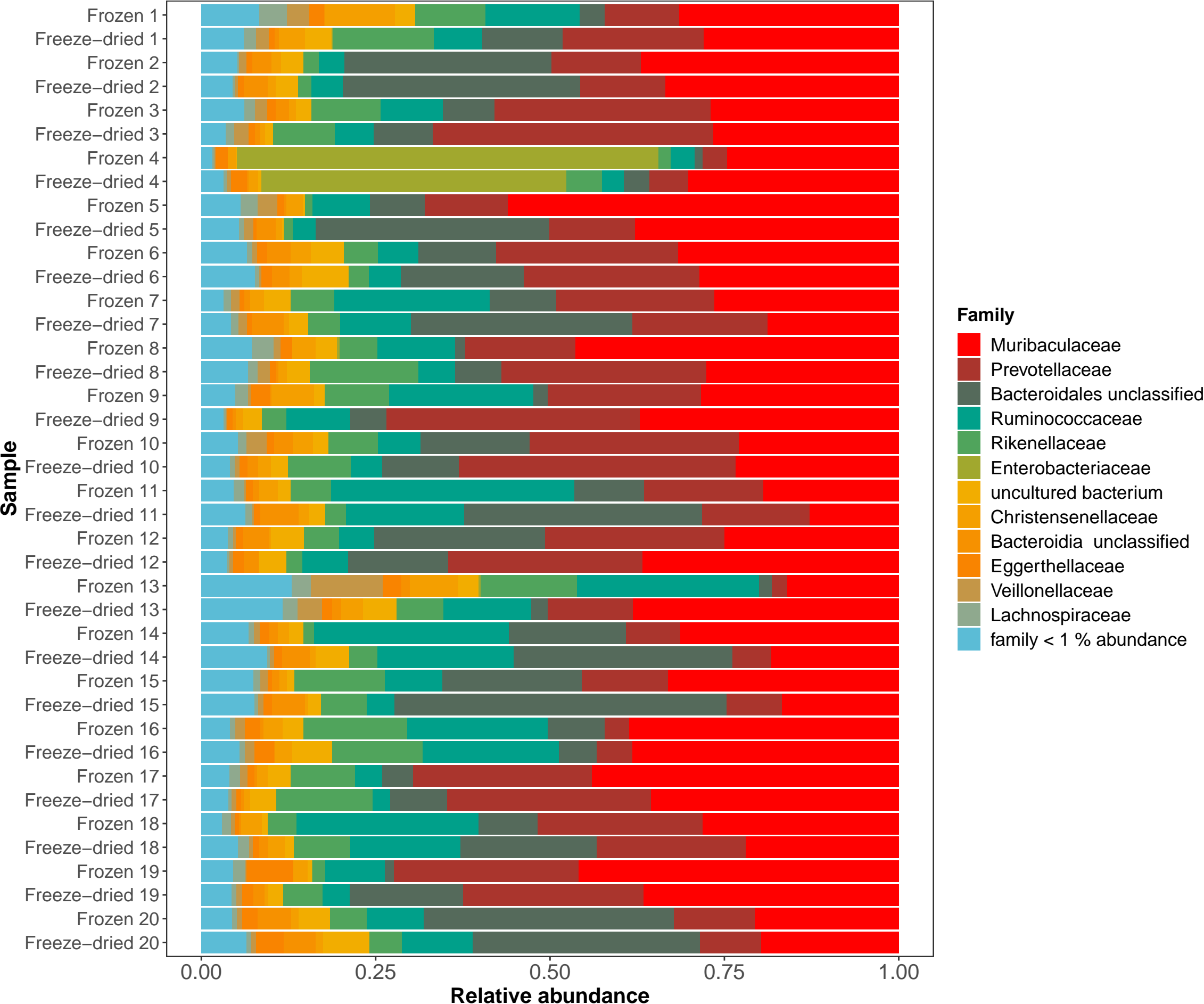

Supplement: Supplemental Information 1 — Sample replicates as either freeze-dried or frozen on y-axis and relative abundance on x-axis. Amplicon Sequence Variants (ASVs) belonging to other families are combined within “family < 1% abundance” and colors explained in legend. [file peerj-10-13095-s001.pdf]

● Freeze-dried ● Frozen

A) Weighted UniFrac

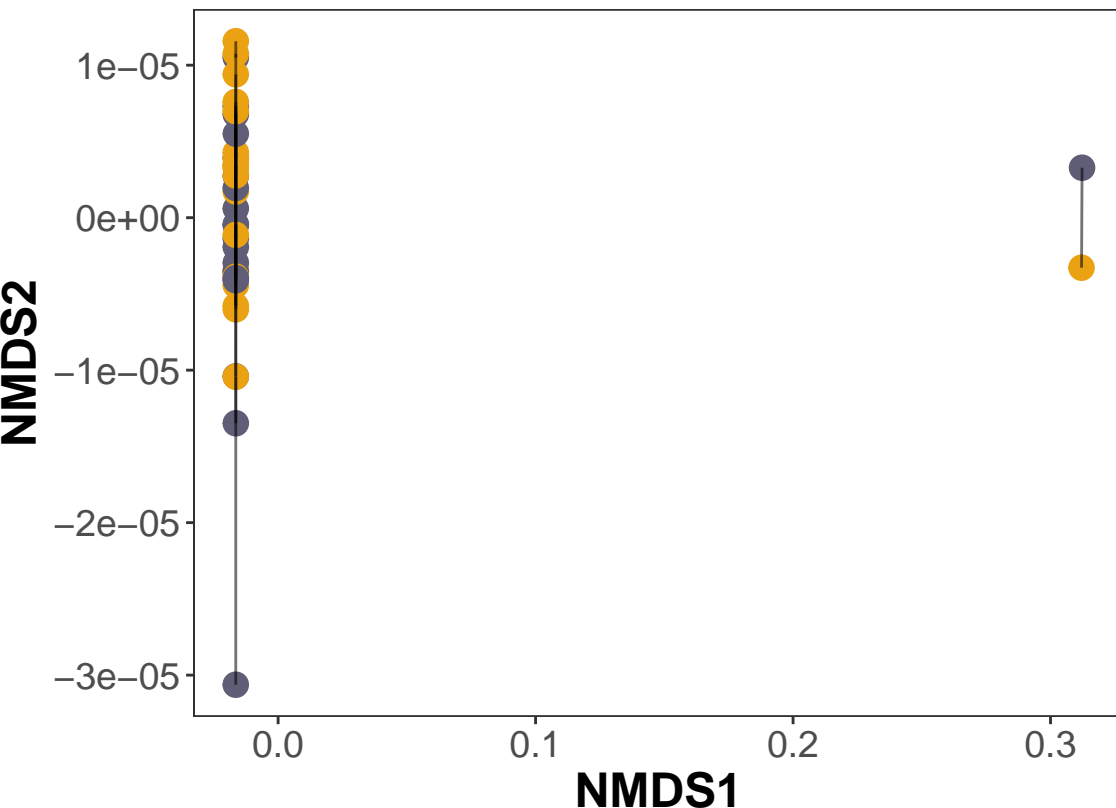

B) Unweighted UniFrac

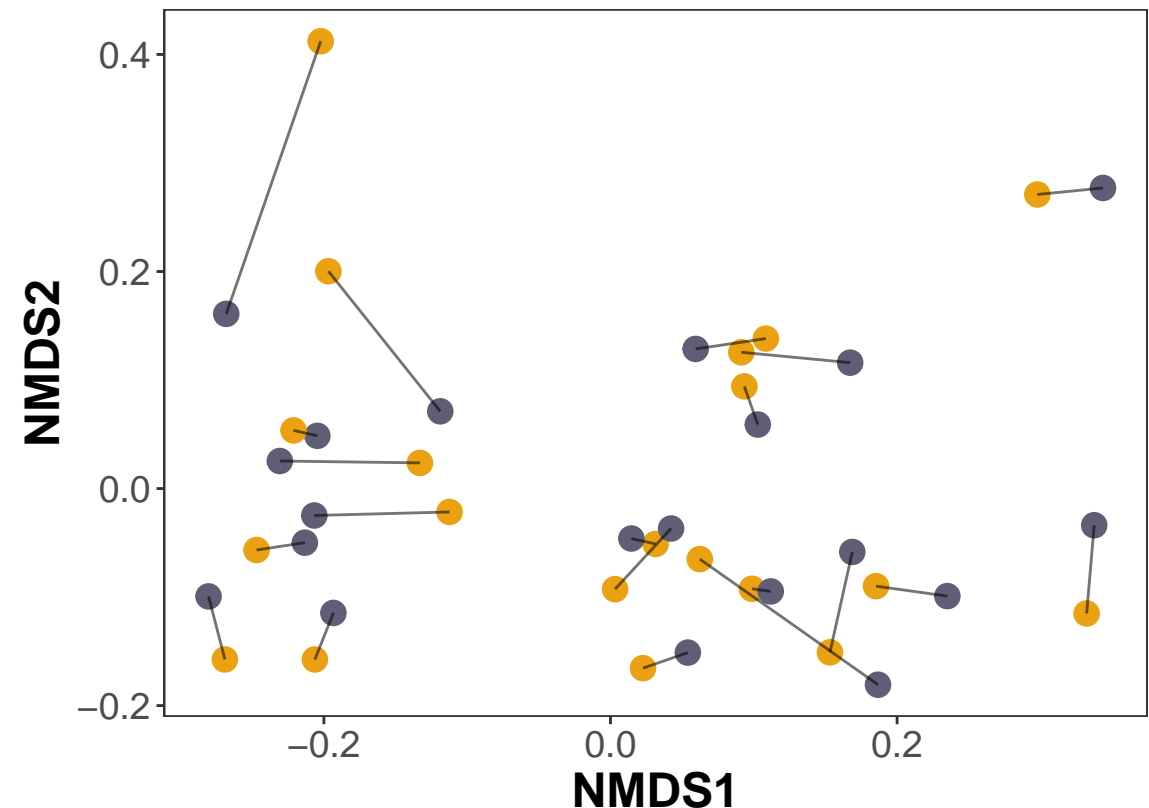

Supplement: Supplemental Information 2 — Non-metric multidimensional scaling (NMDS) on (A) weighted and (B) unweighted unifrac. Stress weighted = 7.705324e−05, unweighted = 0.1652642. Lines between points pair replicates of the same original sample and point colour represent the two sample preservation treatments: freeze-dried (yellow); frozen (blue-grey). [file peerj-10-13095-s002.pdf]

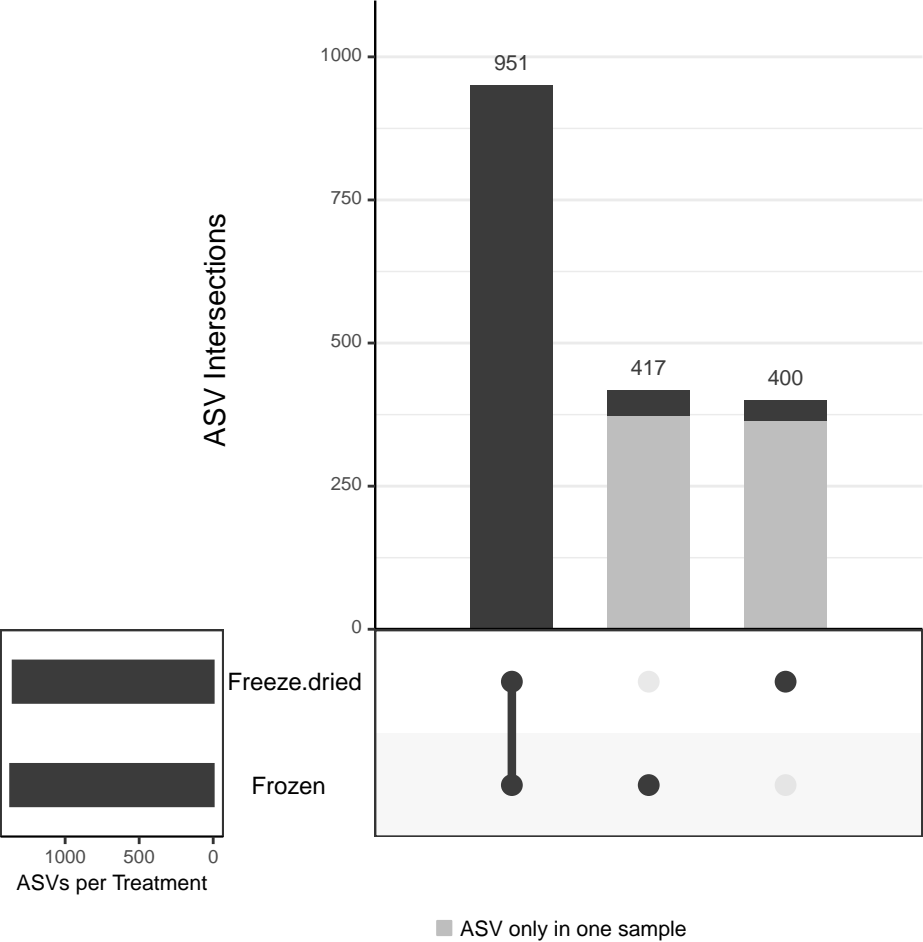

Supplement: Supplemental Information 3 — Out of 1768 ASVs in our data set, 951 were shared between both sample treatments. The two treatments had a similar number of unique ASVs (417 and 400 ASVs). Grey shade of bars represents ASVs unique to a single sample (N = 735). [file peerj-10-13095-s003.pdf]

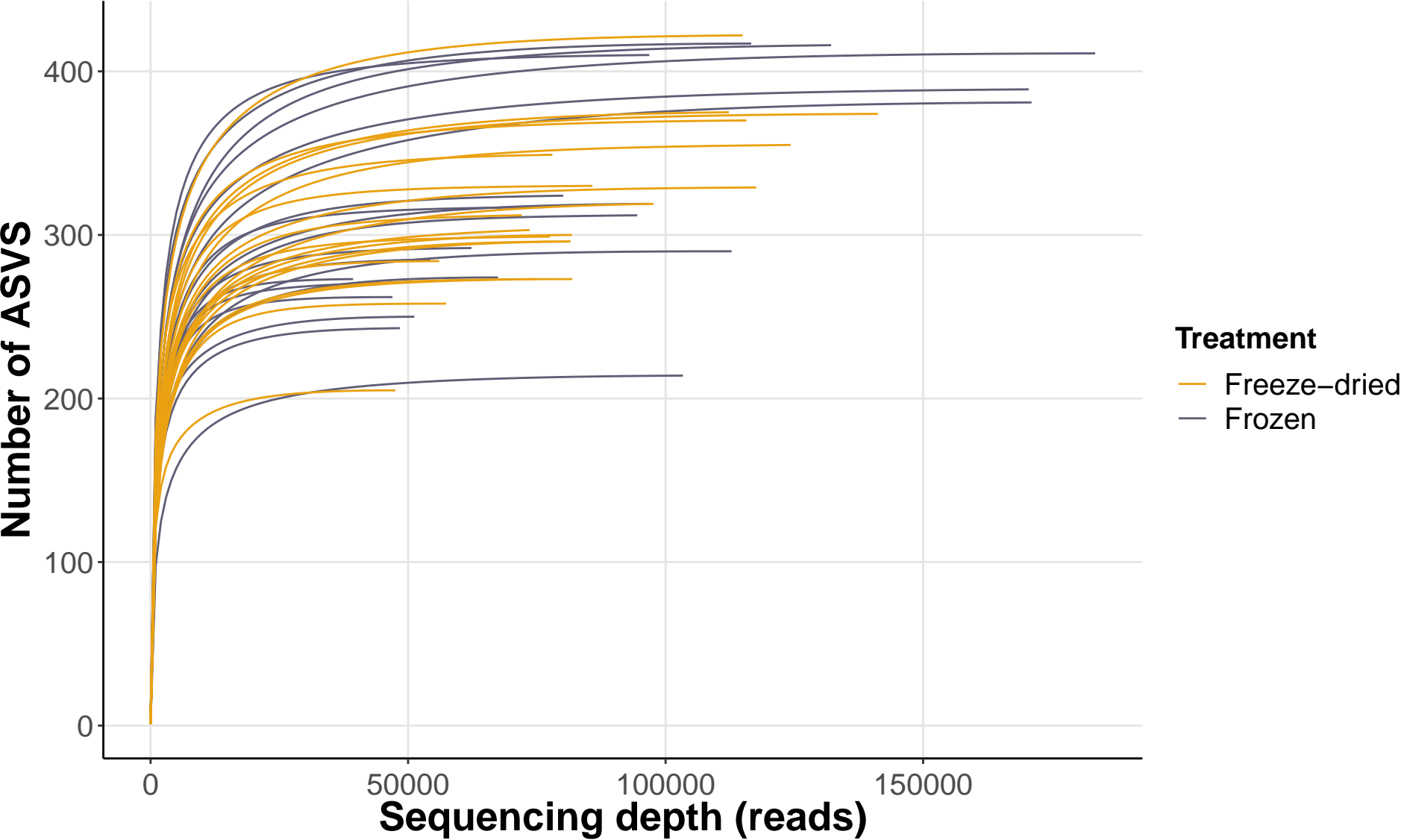

Supplement: Supplemental Information 4 — Sequencing depth is the number of reads within a sample and Number Amplicon Sequence Variants (ASVs) is the number of ASVs detected within the given sample. Colour of line represent the two sample preservation treatments: freeze-dried (yellow); frozen (blue-grey). [file peerj-10-13095-s004.pdf]
