# Supplementary material for: Freeze-drying can replace cold-chains for transport and storage of fecal microbiome samples
Source: PeerJ. 2022 Mar 15;10:e13095. doi: 10.7717/peerj.13095 (PMC8932309; doi:10.7717/peerj.13095)
Supplement: Supplemental Information 5 — p.adjust are p-values adjusted with Bonferroni adjustment for multiple testing. [file peerj-10-13095-s005.docx]

| **phylum - family** | **p** | **p.adjust** |
| --- | --- | --- |
| Firmicutes Christensenellaceae | <0.001 | <0.001 |
| Firmicutes Ruminococcaceae | <0.001 | 0.006 |
| Firmicutes Lachnospiraceae | 0.001 | 0.015 |
| Bacteroidetes Bacteroidales unclassified | 0.004 | 0.063 |
| Actinobacteria uncultured bacterium | 0.009 | 0.142 |
| Proteobacteria uncultured bacterium | 0.009 | 0.142 |
| Cyanobacteria uncultured bacterium | 0.009 | 0.142 |
| Bacteroidetes Bacteroidia unclassified | 0.09 | 1 |
| Bacteroidetes Muribaculaceae | 0.09 | 1 |
| Firmicutes Veillonellaceae | 0.154 | 1 |
| Bacteroidetes Prevotellaceae | 0.216 | 1 |
| Actinobacteria Eggerthellaceae | 0.33 | 1 |
| Proteobacteria Enterobacteriaceae | 0.398 | 1 |
| family < 1 % abundance | 0.812 | 1 |
| Bacteroidetes Rikenellaceae | 0.985 | 1 |
